# Exploring Natural Allelic Variations of the β-Triketone Herbicide Resistance Gene *HIS1* for Application in *indica* Rice and Particularly in Two-Line Hybrid Rice

**DOI:** 10.1186/s12284-020-00448-7

**Published:** 2021-01-07

**Authors:** Qiming Lv, Xiuli Zhang, Dingyang Yuan, Zhiyuan Huang, Rui Peng, Jiming Peng, Zuren Li, Li Tang, Ducai Liu, Xiaomao Zhou, Lifeng Wang, Lang Pan, Ye Shao, Bigang Mao, Yeyun Xin, Lihuang Zhu, Bingran Zhao, Lianyang Bai

**Affiliations:** 1 State Key Laboratory of Hybrid Rice, Hunan Hybrid Rice Research Center, Changsha, China; 2 Longping Branch of Graduate School, Hunan University, Changsha, China; 3 Rice Research Institute, Hunan Academy of Agricultural Sciences, Changsha, China; 4 Hunan Agricultural Biotechnology Research Institute, Hunan Academy of Agricultural Sciences, Changsha, China; 5 Institute of Plant Protection, Hunan Academy of Agricultural Sciences, Changsha, China; 6 Institute of Genetics and Developmental Biology, Chinese Academy of Sciences, Beijing, China

**Keywords:** *indica* rice, Hybrid rice, *HIS1 gene*, β-Triketone herbicides, Benzobicyclon

## Abstract

**Background:**

Benzobicyclon (BBC) is a β-triketone herbicide (bTH) used in rice paddy fields. It has the advantages of high efficiency, low toxicity, high crop safety, and good environmental compatibility, and shows efficacy against paddy weeds resistant to other types of herbicides. However, as some important *indica* rice varieties are susceptible to BBC, BBC is currently only registered and applied in *japonica* rice cultivation areas.

**Results:**

By analyzing haplotypes of the bTHs broad-spectrum resistance gene *HIS1* and phenotypes for BBC in 493 major *indica* rice accessions in China, we identified a novel non-functional allelic variant of *HIS1* in addition to the previously reported 28-bp deletion. Through detection with markers specific to the two non-functional mutations, it was clear that 25.4% of *indica* conventional varieties, 59.9% of fertility restorers, and 15.9% of sterile lines were susceptible to BBC. In addition, due to natural allelic variations of the *HIS1* gene in the sterile and restorer lines, some two-line hybrid sterile lines were sensitive to bTHs, and the corresponding restorers were resistant. We showed the potential effectiveness of using bTHs to address the issue of two-line hybrid rice seed purity stemming from the self-crossing of sterile lines during hybrid rice seed production. Finally, allelic variations of the *HIS1* gene may also play an important role in the mechanized seed production of hybrid rice.

**Conclusions:**

Our findings offer guidance for the application of BBC in *indica* rice areas and provide a non-transgenic approach to address the seed purity issue of two-line hybrid rice.

## Background

Rice is one of the most important crops in the world (Sasaki, and International Rice Genome Sequencing, P 2005). Rice is primarily cultivated in two ways, namely puddled-transplanted rice (TPR) and direct seeding of rice (DSR) (Kumar and Ladha 2011). At present, Asian countries mainly rely on TPR, while some European countries, Australia, and the United States rely almost entirely on DSR (Luo et al. 2019). Although DSR has many advantages, such as water and labor-saving potential and low methane emissions (Farooq et al. 2011; Kumar and Ladha 2011; Rao et al. 2007), it is more susceptible than TPR to weed damage. Yield losses are much higher under DSR than under TPR in the absence of effective weed control options (Mahajan et al. 2013; Rao et al. 2007). In countries where DSR is widely adopted, herbicide use has increased steadily; however, the long-term use of such agents can lead to the emergence of resistant weeds (Farooq et al. 2011; Gould et al. 2018; Powles and Yu 2010). Therefore, the development and application of new types of herbicides are essential for further rice production.

Benzobicyclon (BBC) is a novel paddy-bleaching β-triketone herbicide (bTH) (Komatsubara et al. 2009) that can be applied from pre-emergence to early post-emergence in paddy fields, showing broad-spectrum resistance against annual grasses, sedges, and broadleaf weeds but being harmless to TPR and DSR (Bi et al. 2018; Komatsubara et al. 2009; Maeda et al. 2019). bTHs mainly target 4-hydroxyphenylpyruvate dioxygenase (HPPD), a key enzyme in the biosynthesis of plastoquinone and tocopherol (Kraehmer et al. 2014; Moran 2005). Once the activity of HPPD is suppressed, the normal synthesis pathway of plastoquinone and tocopherol can be blocked, which in turn leads to reduced carotenoid biosynthesis and blocked electron transfer in the photosynthetic chain. Finally, photosynthesis ceases, and plants die due to bleaching (Beaudegnies et al. 2009; Kraehmer et al. 2014). As an herbicide that inhibits HPPD, BBC differs from herbicides that inhibit acetolactate synthetase (ALS) and acetyl-CoA carboxylase (ACCase), which are widely used in paddy fields (Jabusch and Tjeerdema 2005; Kumar and Ladha 2011; Mahajan et al. 2013), and thus BBC can be used as a rotation herbicide in these fields. The number of weed species with resistance to herbicides that inhibit HPPD is minimal (Gould et al. 2018), and BBC shows efficacy against paddy weeds resistant to other types of herbicide, including sulfonylureas (Maeda et al. 2019).

BBC is a selective herbicide. To date, it has only been registered and applied in *japonica* rice cultivation areas because some *indica* rice varieties are susceptible to BBC. Recently, the rice gene *HIS1* (*HPPD INHIBITOR SENSITIVE 1*), which confers resistance to BBC and other bTHs in *japonica* varieties, was identified. *HIS1* encodes a Fe (II)/2-oxoglutarate (2OG)-dependent oxygenase that detoxifies bTHs by catalyzing their hydroxylation (Maeda et al. 2019). Sequence alignment revealed that *his1* in BBC-sensitive varieties harbors a 28-bp deletion in the third exon, which leads to a nonsense mutation (Maeda et al. 2019). PCR-based genotyping showed that the 28-bp deletion was not found in most tested *japonica* rice varieties but was present in some *indica* rice varieties, including Tadukan and IR64, which are backbone parents used in *indica* rice breeding (Huang et al. 2018; Mackill and Khush 2018). Therefore, it can be inferred that many modern *indica* rice varieties are susceptible to BBC due to the nonfunctional *his1* with the 28-bp deletion.

China is one of the main rice-producing countries, with an annual rice cultivation area that exceeds 30 million hm^2^ (Hu et al. 2016). China’s rice cultivation area can be broadly divided into northern *japonica* rice areas and southern *indica* rice areas. The area planted with *indica* rice in China accounts for more than 70% of the total rice area (Wan et al. 2010; Zhang et al. 2016). In view of the excellent performance of BBC in weed control, clarifying which *indica* rice varieties can tolerate this type of herbicide should offer many benefits. The vast majority of hybrid rice varieties are *indica*, and the annual hybrid rice cultivation area exceeds 16.7 million hm^2^ in China due to good yield performance (Hu et al. 2016). In addition, the cultivation area of hybrid rice has also continued to increase in other countries (Xie and Peng 2016). However, although hybrid rice has many advantages, its seed production process is complicated. Hybrid rice parents need to be planted separately, and hybrids and male parents need to be harvested separately, which is time-consuming, laborious, and not suitable for mechanization (Xia et al. 2019). By breeding restorer lines containing herbicide-sensitive genes, the herbicides can be sprayed in the seed production field immediately after pollination, and the male parent can be selectively killed, thereby realizing hybrid rice mixed-sowing and mechanized harvesting. In addition, in the two-line hybrid rice seed production process, the fertility of photoperiod- and thermo-sensitive genic male sterile (P/TGMS) lines is susceptible to temperature, causing the P/TGMS lines to self-cross, thus affecting the purity of the hybrid seed (Ding et al. 2012; Zhou et al. 2012; Zhou et al. 2014). When the purity of the two-line hybrid seed is not sufficient, these hybrid seeds cannot be used in production and will cause significant economic losses. For example, about 6700 ha of two-line hybrid rice failed to produce seed due to the continued low temperature in Jiangsu, Anhui, and Sichuan provinces in China in 2009, which caused a direct economic loss of nearly 100 million Yuan. Furthermore, due to the shortage of hybrid rice seeds, the cultivation area of two-line hybrid rice was reduced by 1.3 million hectares in 2010 (Chen et al. 2011). Through the breeding of P/TGMS lines containing herbicide-sensitive genes, the application of herbicides to hybrid paddy fields at the seedling stage can selectively kill the P/TGMS lines to achieve hybrid purification. There have been many attempts to use herbicides in hybrid rice seed production and hybrid purification, and these attempts have mainly relied on mutagenic and transgenic approaches (Komatsubara et al. 2009; Pan et al. 2006; Shoba et al. 2017; Xia et al. 2019; Zhang et al. 2002; Zhang et al. 2014). However, mutagenesis can only create a few specific materials, and its application is limited. Transgenic rice is not commercially planted in China. The direct selection of materials with different responses to herbicides among the existing hybrid rice parents is likely to play an important role in the further development of hybrid rice.

The aim of this work was to evaluate the potential applications of bTHs in *indica* rice. We performed haplotype analysis of the *HIS1* gene in more than 600 *indica* rice accessions, including hybrid rice parents commonly used in China. In addition to the previously reported 28-bp deletion, we also discovered a novel mutation that caused a loss of function of the *HIS1* gene, and we further clarified which *indica* rice varieties can tolerate bTHs through specific marker detection. We also provided potential applications of bTHs for *indica* cultivation areas and hybrid rice seed production and purification.

## Results

### Detection of the 28-Bp Deletion and Phenotyping of *indica*

Based on the reported 28-bp deletion that leads to different reactions to BBC among rice cultivars (Maeda et al. 2019), an InDel marker was developed to detect this variation in 631 *indica* accessions commonly used in rice breeding in China (Table S1, Figure S1). The results showed that the 28-bp deletion was widely distributed among China’s *indica* accessions, with an average rate of 28.1%. Among them, the deletion rate of the 3-line restorer was the highest, reaching 50.7%, whereas no deletion was found in the 87 3-line cytoplasm male sterile (CMS) lines. Forty (18.1%) conventional lines, 24 (40.7%) 2-line restorers, and five (9.8%) 2-line genic male sterile (P/TGMS) lines harbored the 28-bp deletion (Table 1). In general, there were more than 28-bp deletions in the restorer lines (48.5%), followed by conventional rice and then the sterile lines (3.6%).

**Table 1:** *Indica* accession numbers of the 28-bp deletion, T1510G mutation, and lines susceptible to BBC

	Total	28-bp deletion	HS	MS	T1510G
Conventional varieties	221	40 (18.1%)	55 (24.9%)	1 (0.5%)	16 (7.2%)
3-line restorer	213	108 (50.7%)	129 (60.6%)	3 (1.4%)	24 (11.3%)
2-line restorer	59	24 (40.7%)	29 (49.2%)	2 (3.4%)	7 (11.9%)
CMS	87	0	NA	NA	14 (16.1%)
P/TGMS	51	5 (9.8%)	NA	NA	3 (5.9%)
Total	631	177 (28.1%)	NA	NA	64 (10.1%)

*CMS* 3-line cytoplasm male sterility, *P/TGMS* Photoperiod- and thermo-sensitive genic male sterile, *HS* Highly susceptible, *MS* Moderately susceptible, *NA* No phenotype

**Fig. 1:**
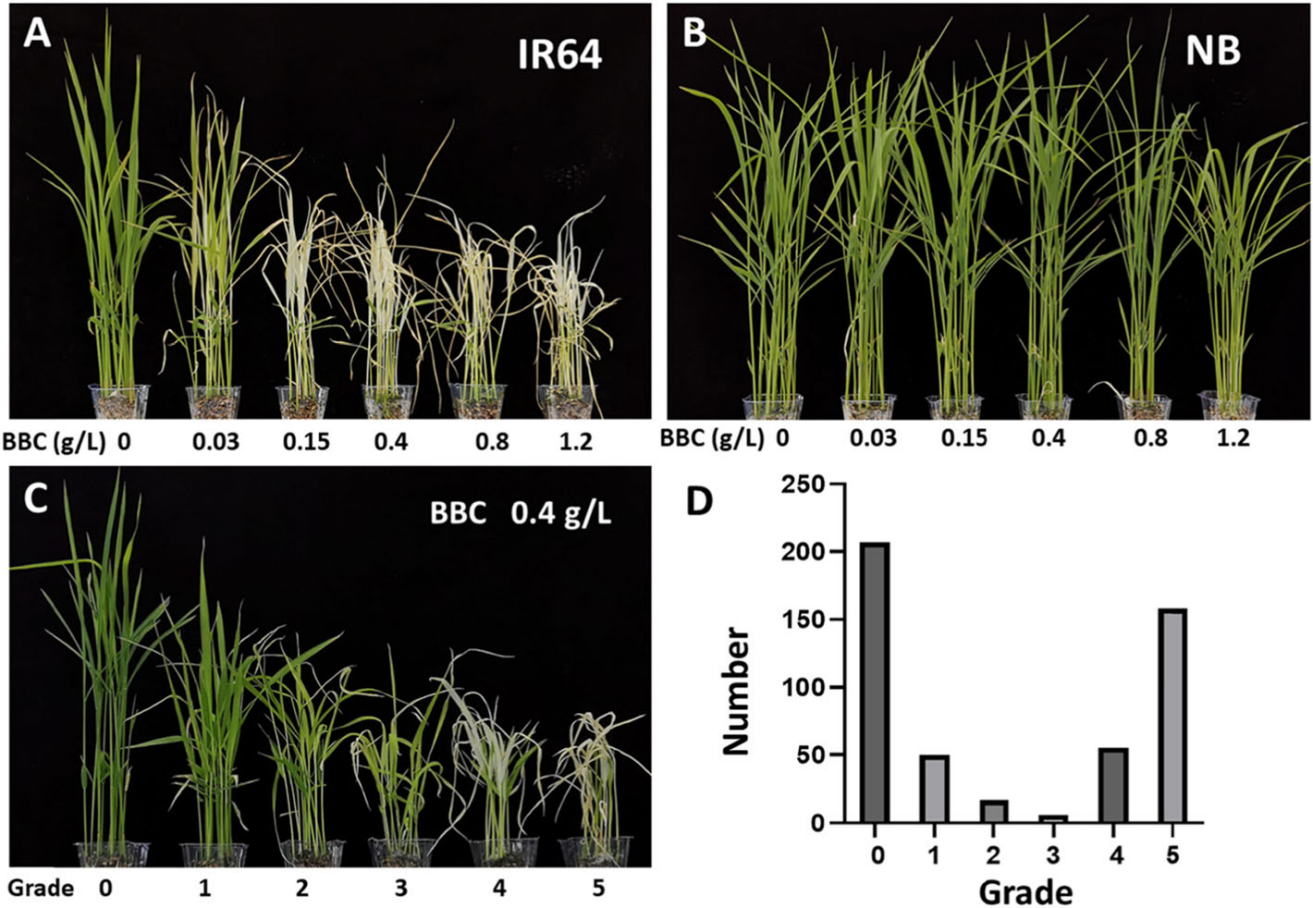
Phenotyping of *indica* rice accessions. **a** Concentration-dependent effects of BBC on IR64. **b** Concentration-dependent effects of BBC on Nipponbare (NB). **c** Different graded phenotypes after treatment with 0.4 g/L BBC. **d** Number of accessions with different graded phenotypes. Grade 0: unaffected seedling; grade 1: seedling growth was slightly weakened; grade 2: seedlings with an obvious dwarfing phenomenon; grade 3: seedlings without obvious bleaching but where some had started to die; grade 4: seedlings bleached completely; grade 5: seedlings completely died

In order to determine whether the BBC-sensitive phenotype is completely related to the 28-bp deletion, we selected 493 accessions including conventional and restorer lines (sterile line seeds are more valuable, and we did not have enough seeds) for BBC application. At first, Nipponbare (NB) (*HIS1*) and IR64 (*his1*) were used to determine suitable BBC concentrations. The results showed that IR64 started to show signs of injury when the concentration was 0.03 g/L. When the concentration reached 0.15 g/L, the difference between IR64 and NB was already obvious, and when the concentration reached 1.2 g/L, NB also started to show susceptibility (Fig. 1a, b). Ultimately, a concentration of 0.4 g/L was selected to evaluate the resistance of *indica* accessions to BBC. According to the different responses of rice to BBC, we divided the phenotypes into six grades: grades 0 and 1 showed complete resistance, grades 2 and 3 were intermediate types, and grades 4 and 5 were completely sensitive (Fig. 1c). The BBC spraying results showed that most *indica* accessions showed high resistance or high sensitivity, and only a few were of the intermediate type, which is probably related to the *HIS1* gene as the major BBC resistance gene (Maeda et al. 2019) (Fig. 1d). Among the three different types of rice accessions, the BBC-sensitive trait had a certain relationship with the 28-bp deletion, but the number of BBC-sensitive accessions obviously exceeded the number of 28-bp deletions (Table 1). Therefore, in addition to the 28-bp deletion, there may be other mutation(s) of the *HIS1* gene that lead to BBC sensitivity.

### The T1510G Mutation in the *HIS1* Gene Leads to the Loss of Function

**Fig. 2:**
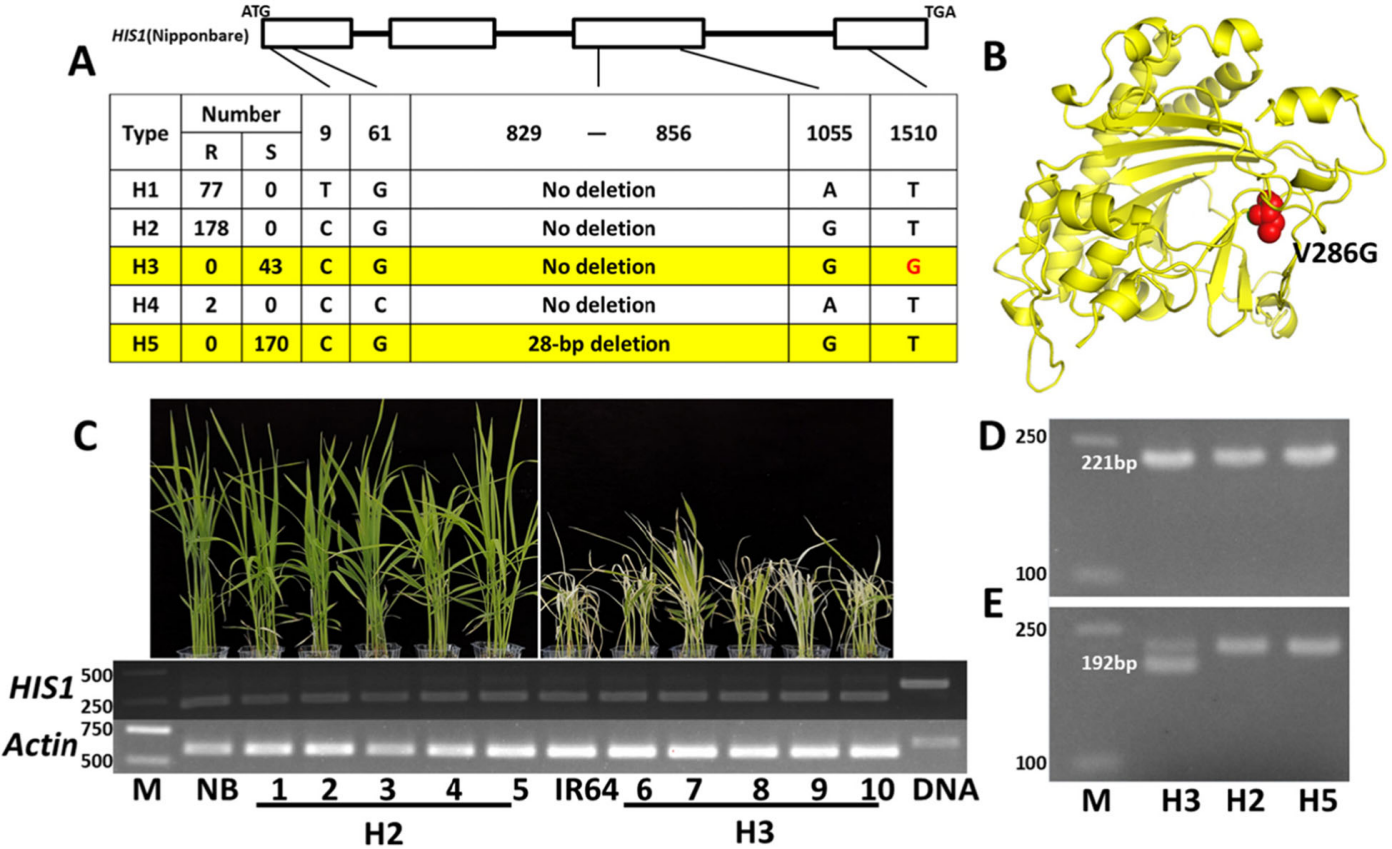
T1510G mutation leads to the loss of function of *HIS1.* **a** Different haplotypes of *HIS1* in the 493 *indica* rice accessions. R, highly resistant to BBC without moderate resistance. S, highly susceptible to BBC without moderate susceptibility. **b** Three-dimensional structure model of HIS1 and the location of the V286G (T1510G) mutation (red). **c** RNA expression of *HIS1* in some accessions harboring H2 and H3. 1, Nongxiang32; 2, Guichao2hao; 3, Shuhui257; 4, Minghui70; 5, Youzhan; 6, Xianhui207; 7, IR50; 8, Aizaizhan; 9, Xiangwanxian6hao; 10, R898; DNA, Nipponbare genomic DNA. **d**, **e** dCAPS marker designed for the T1510G mutation. **d** and **e** represent the PCR products before and after treatment with the restriction enzyme *Dde1*. M, DNA marker

In order to determine whether BBC sensitivity is caused by mutations of the *HIS1* gene, we performed PCR cloning and sequencing of the *HIS1* gene in 493 phenotyped *indica* accessions. The sequencing results showed that there were only five mutation sites in the exons of *HIS1*, which divided the *HIS1* gene into five haplotypes (Fig. 2a), among which H2 and H5 are alleles of the *HIS1* gene in Nipponbare and IR64, respectively. Compared with H2, H1 had two mutations (C9T, G1055A), H3 had only one mutation (T1510G), H4 had two mutations (G61C, G1055A), and the 28-bp deletion was found only in H5. When the phenotype of each *indica* accession was correlated with the haplotype of the *HIS1* gene, we found that accessions containing H1, H2, and H4 showed full resistance to BBC, while H3 and H5 showed complete susceptibility to BBC (Fig. 2a). Studies have shown that non-functional H5 is related to the 28-bp deletion (Maeda et al. 2019). As H3 had only one single base mutation compared with functional H2, it was initially believed that this T1510G mutation was the cause of the loss of function.

The T1510G mutation in H3 caused an amino acid change (V286G) at the protein level (Figure S2), located in the region of the putative substrate pocket that is very important for the function of Fe (II)/2OG-dependent oxygenases (2OGD) (Maeda et al. 2019) (Fig. 2b). After amino acid sequence alignment of 91 experimentally characterized 2OGDs in plants and 114 putative 2OGDs in rice (Kawai et al. 2014), respectively, it was found that this site located in the most conservative motif, and amino acids Val and Leu were dominant at this site (Figure S3). In order to exclude the possibility that non-functional H3 was due to different expressions of *HIS1*, we randomly selected five accessions harboring H2 and H3 to detect the *HIS1* mRNA level, using NB and IR64 as controls. The results showed that *HIS1* was normally expressed in all accessions with H3, and there was no obvious correlation between *HIS1* gene expression and anti-susceptibility to BBC (Fig. 2c). Moreover, through qRT-PCR detection, it was also found that there was no significant difference in the expression levels of each *HIS1* haplotype (Figure S4). An F2 population of Nongxiang42 (containing H2) and Xianhui207 (containing H3) was constructed to analyze the relationship between susceptibility to BBC and the T1510G mutation. The results of spraying BBC on the F2 population showed that among the 968 individual plants, 737 individual plants showed resistance, and 231 individual plants showed sensitivity to BBC. The ratio of resistant and sensitive plants showed a 3:1 separation ratio (X^2^ = 0.607 < X_(0.05)_^2^ = 3.84), which demonstrated that there was only one dominant resistance gene in Nongxiang42. Next, we designed a dCAPS marker based on the T1510G mutation to distinguish H3 from other haplotypes (Fig. 2d, e). Using this H3-specific marker to detect individual plants of the F2 population, we found that the T1510G mutation was completely co-segregated with susceptibility to BBC (Figure S5). In summary, our results showed that the T1510G mutation caused the loss of function of the *HIS1* gene.

### Natural Allelic Variations of *HIS1* in Cultivated Rice

In order to fully evaluate which *indica* rice accessions can tolerate BBC, we tested all P/TGMS and CMS lines with the H3-specific marker. The results showed that there were three P/TGMS and 14 CMS lines that harbored the T1510G mutation (Table 1). By combining the detection results of the 28-bp deletion in the early stage, we concluded that about 40% of the *indica* rice materials and about 25% of conventional rice should be sensitive to BBC (Table 1). Although the proportion of 3- and 2-line restorers that are sensitive to BBC was very high (60% and 52.6%, respectively) due to the low proportion of P/TGMS and CMS lines, the ratio of the *indica* hybrids that are sensitive to BBC should be very low, considering that *HIS1* is a major dominant resistance gene.

**Fig. 3:**
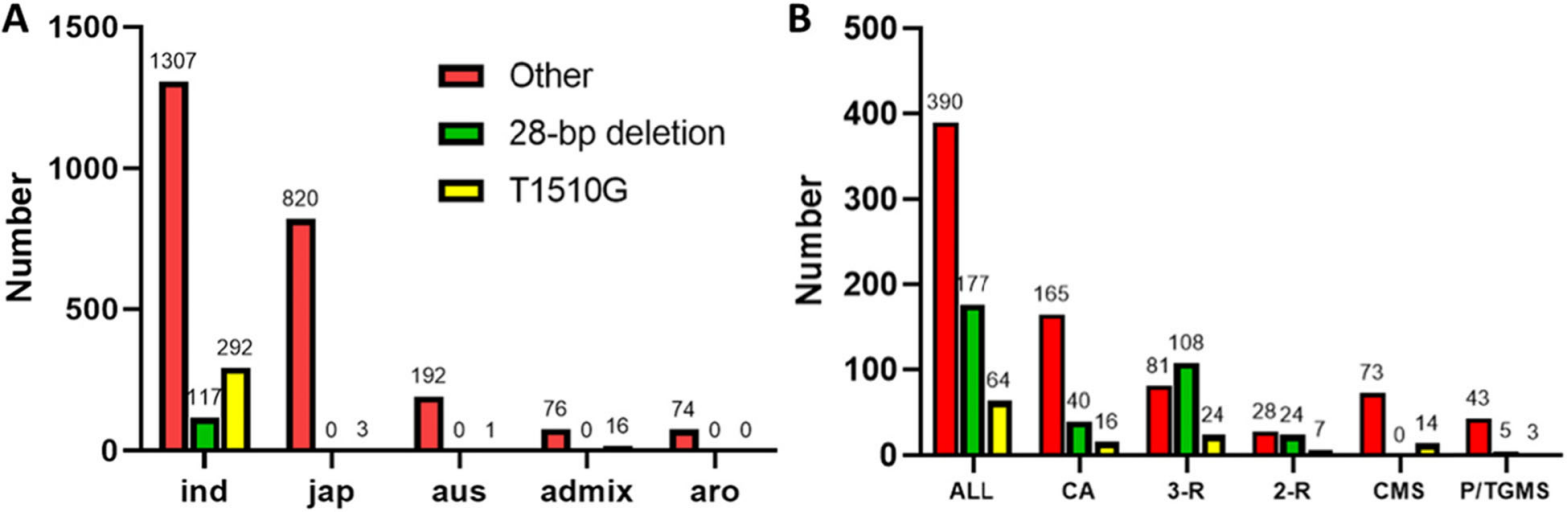
Comparison of the non-functional alleles of *HIS1* in different rice accessions. **a** Numbers of non-functional alleles of *HIS1* in 3000 rice accessions. Ind, *indica*; jap, *japonica*; aus, *aus* accessions; aro, *aromatic* accessions; admix, all other unassigned accessions. **b** Numbers of non-functional alleles of *HIS1* in *indica* rice accessions collected in this study. ALL, all *indica* accessions; CA, conventional accessions; 3-R, 3-line restorer; 2-R, 2-line restorer; CMS, 3-line cytoplasm male sterile line; P/TGMS, photoperiod- and thermo-sensitive genic male sterile

In order to clarify the distribution of non-functional *HIS1* alleles in cultivated rice, we used the 3000 sequenced rice genomes (Wang et al. 2018) to conduct a comprehensive analysis of the 28-bp deletion and T1510G mutation of the *HIS1* gene. The results showed that among 1716 *indica* rice accessions, 117 harbored the 28-bp deletion, and 292 accessions contained the T1510G mutation; among 825 *japonica* rice accessions, no 28-bp deletion was found, and only three accessions had the T1510G mutation. Non-functional alleles of the *HIS1* gene are rarely found in *japonica* rice, and this phenomenon could explain why the BBC herbicide is registered and can be used safely in the *japonica* rice area. In the two other types of cultivated rice, *aus* and *aro*, there was no 28-bp deletion, and only one T1510G mutation was found in *aus* (Fig. 3a). Unlike the 3000 rice accessions, which are mainly landraces with wide genetic variations (Wang et al. 2018), the 631 *indica* accessions we tested were mainly Chinese *indica* rice breeding materials. Non-functional alleles of *HIS1* increased significantly in our 631 *indica* accessions, especially in the restorer lines (Fig. 3b), compared with the 3000 rice accessions, which is likely the result of artificial selection during the rice breeding process.

### BBC Is Applicable for Two-Line Hybrid Rice Seed Purification

**Fig. 4:**
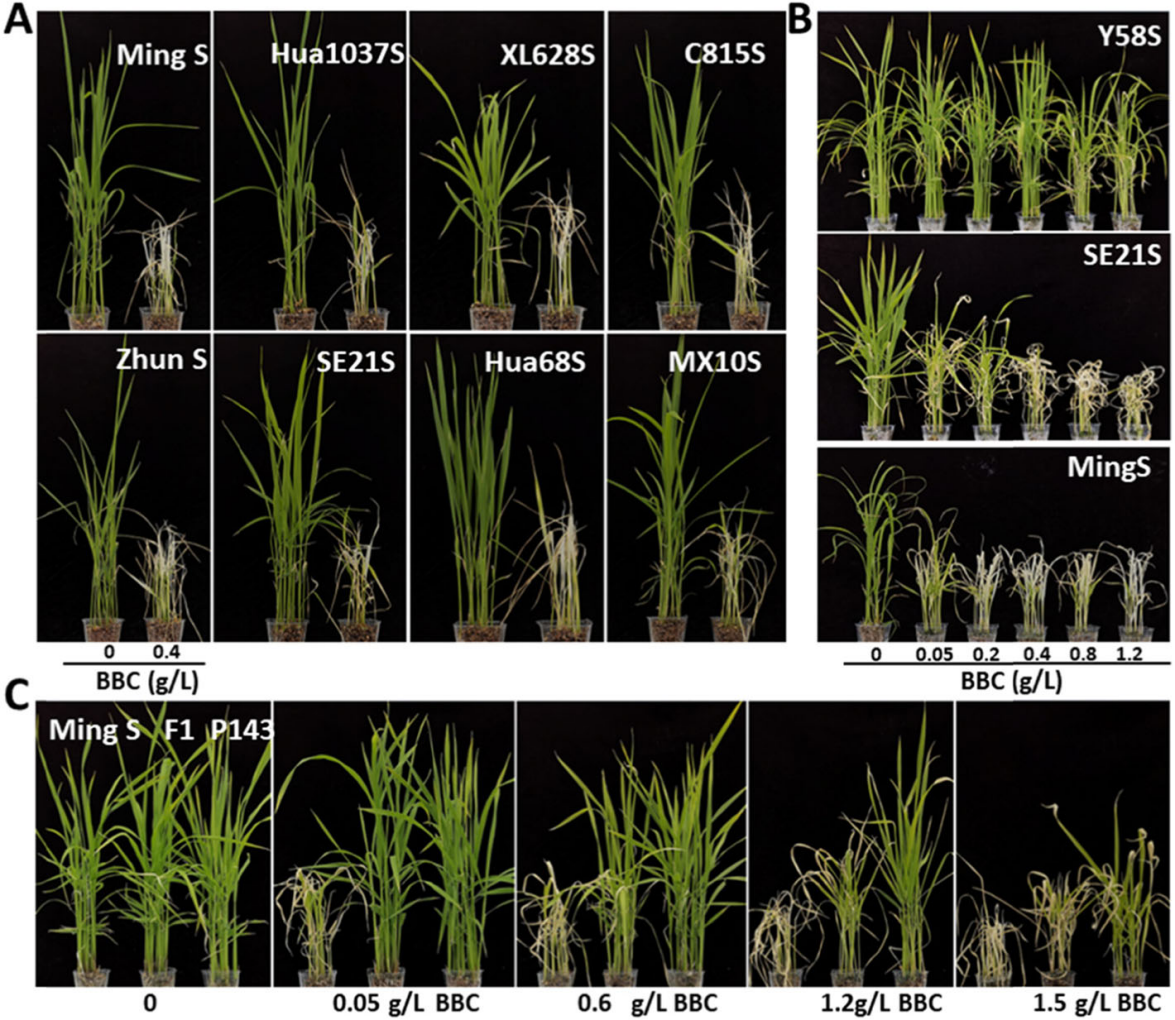
Potential applications of BBC in improving the seed purity of 2-line hybrid rice. **a** Some 2-line P/TGMS are susceptible to BBC. **b** Concentration-dependent effects of BBC on three representative 2-line P/TGMS accessions, Y58S (*HIS1*), SE21S (*his1*-T1510G), and MingS (*his1*–28-bp deletion). **c** Concentration-dependent effects of BBC on the 2-line hybrid variety Mingliangyou143 (F1), female parent MingS, and male parent P143

In order to evaluate the possibility of using the *HIS1* gene to address 2-line hybrid rice seed purity issues, we sprayed BBC on the eight P/TGMS lines that harbored the 28-bp deletion or the T1510G mutation. The results showed that all eight P/TGMS lines were susceptible to BBC (Fig. 4a). In addition, a BBC concentration gradient experiment was conducted on P/TGMS lines SE21S (T1510G) and MingS (28-bp deletion) with Y58S as a control. The results showed that SE21S and MingS were injured at a low BBC concentration (0.05 g/L), while Y58S exhibited no obvious damage until the BBC concentration reached 1.2 g/L (Fig. 4b). Finally, we chose a commercialized 2-line hybrid rice variety Mingliangyou143 from China, of which the female and male parents are MingS and P143 (containing *HIS1* H2), respectively, to examine the effect of BBC on the purification of 2-line hybrid rice seeds. The results showed that at least in the range of 0.05–0.6 g/L, BBC was very effective in killing the female parent MingS but was harmless to the Mingliangyou143 hybrid (Fig. 4 C). Moreover, it was found that BBC could kill the P/TGMS line without affecting the hybrid when a small amount of MingS was mixed into Mingliangyou143 (Figure S6). At high concentrations, the hybrid containing the heterozygous *HIS1/his1* gene appeared to be less resistant to BBC than the homozygous male parent, and there were sufficient BBC concentration intervals for the purification of the 2-line hybrid seeds.

**Fig. 5:**
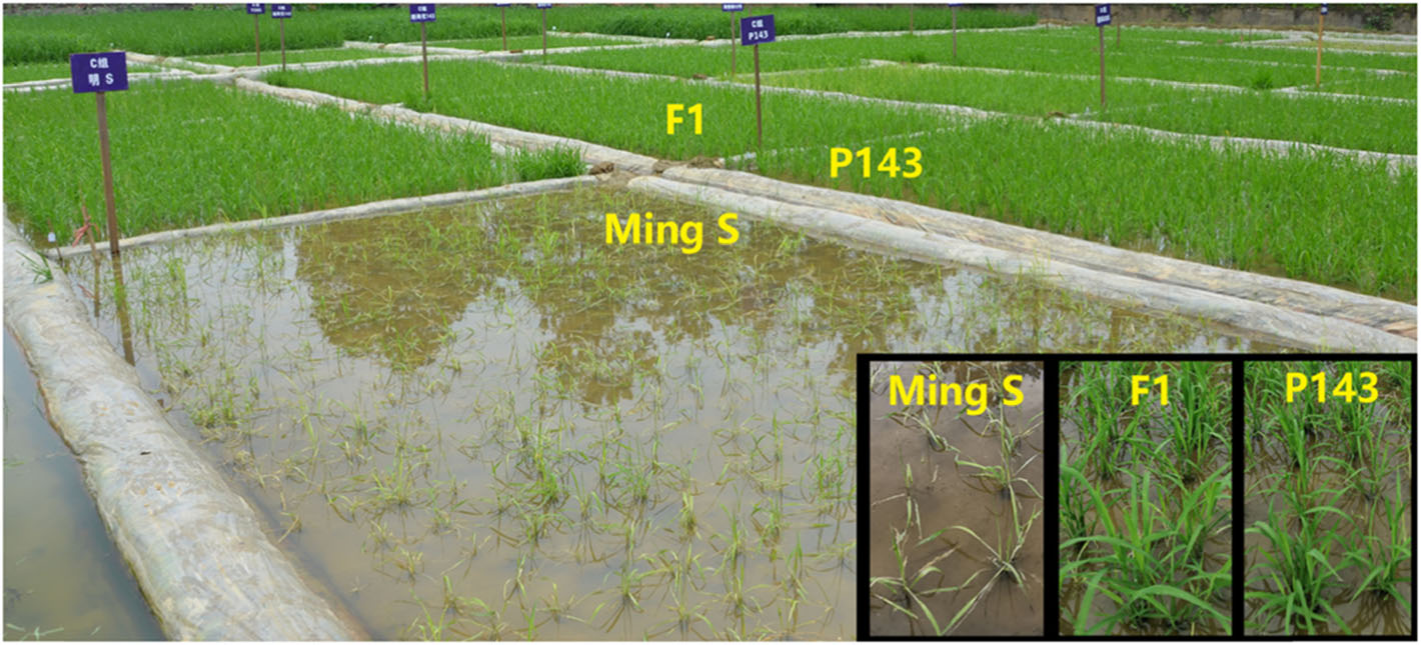
Field phenotype of Mingliangyou143 and its parents to BBC. The 2-line hybrid rice variety Mingliangyou143 (F1) and its female parent MingS and male parent P143 were grown in a paddy field treated with BBC (300 g of active ingredient/ha) at 10 days after direct seeding. Examination of the plants at 25 days after direct seeding revealed that F1 and P143 were resistant to BBC, whereas MingS was sensitive

To determine whether BBC could be used to address 2-line hybrid rice seed purity issues in the field, we sprayed BBC on Mingliangyou143 and its parents in the field. We found that under direct seeding cultivation, when the BBC dosage was 300 g/ha (the recommended dosage is 150–225 g/ha for weeding in the field), all seedlings of the female parent MingS showed obvious bleaching, and growth stopped completely. By contrast, Mingliangyou143 and the male parent P143 grew normally (Fig. 5). These results demonstrated that BBC could address the problem of 2-line hybrid rice seed purity during weed control in the field.

### Potential Applications of Other β-Triketone Herbicides

**Fig. 6:**
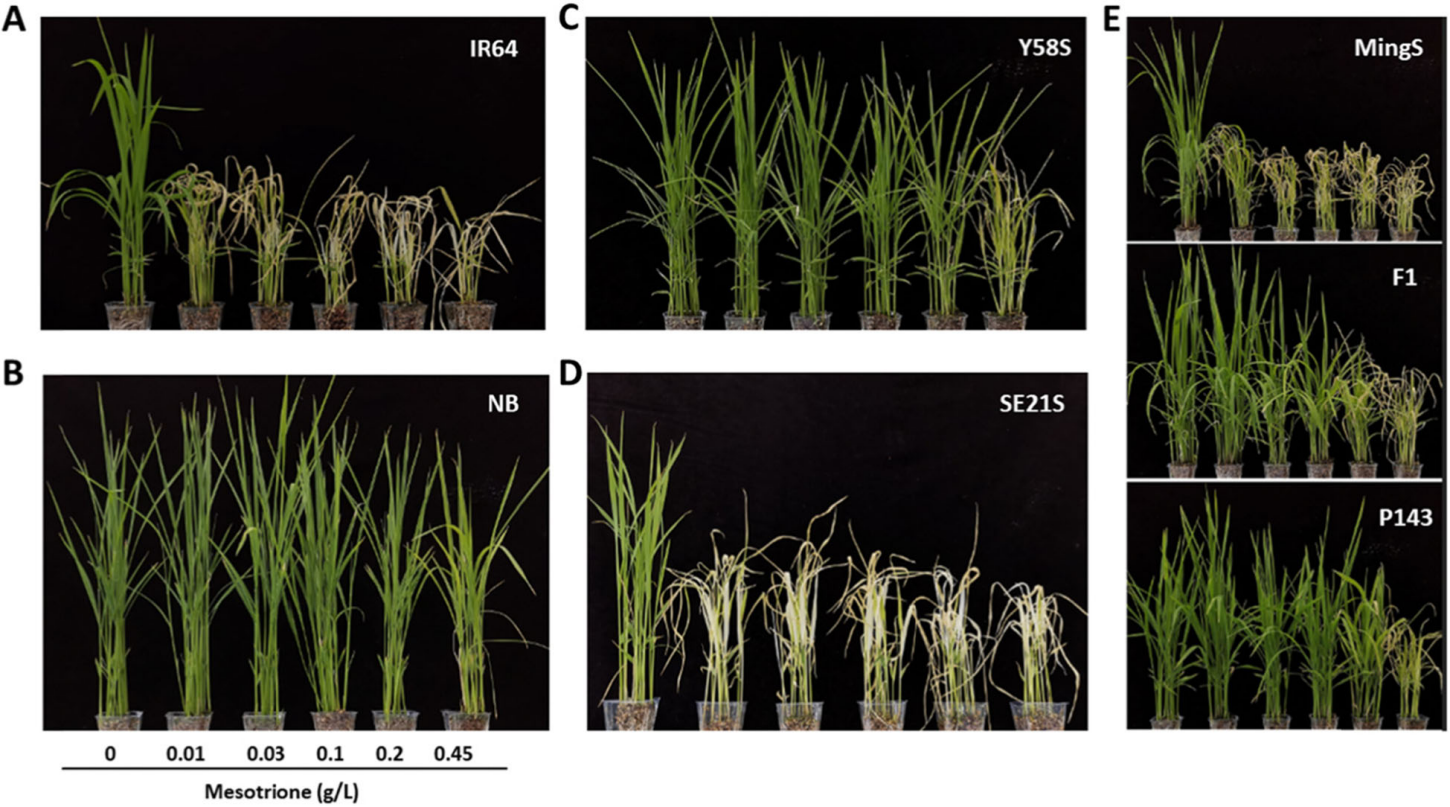
Potential applications of mesotrione in improving the seed purity of 2-line hybrid rice. **a**–**d** Concentration-dependent effects of mesotrione on IR64, Nipponbare (NB), Y58S, and SE21S. **e** Concentration-dependent effects of mesotrione on the 2-line hybrid variety Mingliangyou143 (F1), female parent MingS, and male parent P143

The commonly used bTHs include BBC, mesotrione, sulcotrione, tembotrione, and tefuryltrione. Currently, mesotrione is the most widely used HPPD-inhibitor herbicide globally (Hua 2015). In order to identify whether other bTHs are the same as BBC, i.e., can be used in *indica* rice areas and 2-line hybrid rice seed purification, we chose mesotrione for further study. As shown in Fig. 6a–d, IR64 and SE21S started to become susceptible to mesotrione when the concentration exceeded 0.01 g/L. However, NB and Y58S only started to show obvious injury when the mesotrione concentration reached 0.45 g/L. In addition, the spraying of a mesotrione concentration gradient on Mingliangyou143 and its parents showed that mesotrione could also be used for the purification of 2-line hybrid rice seed, at least when the application concentration ranges from 0.01 g/L to 0.1 g/L (Fig. 6e). Since the *HIS1* gene has been confirmed to be a broad-spectrum resistance gene for bTHs (Maeda et al. 2019) and mesotrione is similar to BBC in terms of the phytotoxicity responses of different *indica* rice varieties, we determined that by detecting the *HIS1* gene in *indica* rice, there is potential to use bTHs in *indica* rice areas and in the purification of 2-line hybrid rice seeds.

## Discussion

### Potential Applications of BBC in *indica* Rice Areas

bTHs are HPPD inhibitors that are applied widely in crops (Kraehmer et al. 2014). These inhibitors exhibit various advantages, including their high efficiency, low toxicity, safe application in crops, effectivity against resistant weeds, low environmental impact, and low impacts on subsequent crops. As a bTH herbicide used in paddy fields, BBC is currently only registered and applied in *japonica* rice cultivation areas. Through analysis of the non-functional mutations of the *HIS1* gene in 3000 rice accessions, we found that there was almost no non-functional *HIS1* allele in *japonica* rice; this is consistent with the fact that BBC can be registered and applied in *japonica* rice areas. In addition, in the *aus* and *aro* rice accessions, the ratio of non-functional *his1* was also very low, and thus the application of BBC may be potentially promising in these two types of rice cultivation areas. In cases where some important *indica* rice varieties are BBC-sensitive, it is important to determine the application scope of BBC in *indica* rice areas. This would allow BBC to be applied to a large area, which could resolve the issue of the resistance of weeds to other types of herbicide, including sulfonylureas (Maeda et al. 2019). In this study, by sequencing the bTHs broad-spectrum resistance gene *HIS1* in 493 *indica* rice accessions commonly used as conventional lines and restorers of hybrid rice in China, a total of five haplotypes of *HIS1* were identified. Among them, H1, H2, and H4 were resistant to BBC, while H3 and H5 were non-functional. The loss of function of H5 was due to the previously reported 28-bp deletion in the third exon, while the non-functional H3 was found to be caused by the T1510G mutation in the fourth exon in this study. Two markers specific to the 28-bp deletion and the T1510G mutation were designed to test the *indica* rice accessions. Ultimately, we determined that approximately 25% of conventional rice, 15% of the sterile lines, and 60% of the restorer lines harbored the two non-functional mutations in *HIS1*. Considering that the *HIS1* gene is a major dominant resistance gene, hybrid rice varieties containing heterozygous *HIS1/his1* could be resistant to bTHs. Therefore, although the ratio of non-functional *HIS1* alleles was much higher in the restorer lines compared to the other types of rice, the ratio of hybrid rice varieties susceptible to bTHs should be relatively low. In addition, through concentration gradient experiments, it was found that there was no particularly significant difference between resistant *indica* and *japonica* rice. Therefore, as rice cultivation in China has largely begun to be transformed into agricultural cooperatives and large grain farms, it would be possible to apply BBC herbicides in specific *indica* rice areas in the future.

### Potential Applications of bTHs in Seed Purification of 2-Line *indica* Hybrid Rice

At present, *indica* rice areas in China are mainly planted with hybrid rice, with an annual planting area of 16 million hm^2^, of which 2-line hybrid rice accounts for more than 30% (Hu et al. 2016). In addition, in other countries, such as the United States, 2-line hybrid rice is also being planted on a large scale (Xie and Peng 2016). Although 2-line hybrid rice has the advantages of simpler breeding procedures and more freedom in combination, the P/TGMS sterile lines are negatively affected by light and temperature during seed production, resulting in self-crossing, which seriously affects the quality of hybrid seeds. Previous studies have attempted to create herbicide-sensitive sterile lines or herbicide-resistant restorer lines through genetic modification and physical/chemical mutagenesis (Shoba et al. 2017; Tang et al. 2020; Xia et al. 2019; Zhang et al. 2002; Zhang et al. 2014), but have not resolved the seed purity problem of 2-line hybrid rice at a large scale except for the use of imidazolinone-resistant restorers in the United States (Sudianto et al. 2013). In this study, we identified eight 2-line sterile lines naturally sensitive to bTHs, whereas many of the corresponding restorer lines were resistant. Therefore, in this two-line hybrid rice, bTHs can be used directly to purify hybrid rice seeds. Moreover, we found that mesotrione required a lower lethal dose and had lower costs than BBC for seed purification. Since the *HIS1* gene has no other side effects on rice growth and yield (Maeda et al. 2019), it would be possible to introduce *his1* into 2-line sterile lines and *HIS1* into restorer lines in future research to control against the risks of 2-line hybrid rice seed purity.

### Potential Applications of bTHs in the Mechanized Seed Production of *indica* Hybrid Rice

By comparing the ratio of non-functional *HIS1* alleles in the *indica* accessions of the 3000 rice accessions and in commonly used *indica* rice in China, we found that the percentage of non-functional alleles of the *HISI* gene in breeding materials in China, especially in the restorer lines, was much higher. By analyzing each material, we found that this increased percentage might be due to some important backbone restorer lines, such as IR24, IR26, Minghui63, Shuhui527, and Mianhui725 (supplementary Table 1), all of which contained the non-functional alleles of *HIS1*. Considering there are many bTH-sensitive restorer lines and bTH-resistant sterile lines, mixed seeding and mechanized harvesting are feasible in the seed production of some hybrid rice varieties, as bTHs can be sprayed after pollination to kill the male parent, leaving only real hybrids. In addition, it is possible that the use of bTHs causes the incomplete killing of restorers after pollination (Tang et al. 2020). This method can therefore also be combined with other hybrid rice mechanized seed production methods, such as the use of husk color differences, grain size differences, or female sterile material systems (Tang et al. 2020; Xia et al. 2019).

## Conclusions

Both the T1510G mutation and the previously reported 28-bp deletion caused the loss of function of the *HIS1* gene. Approximately 75% of conventional *indica* rice varieties and most *indica* hybrid rice varieties in China are able to tolerate bTHs. Allelic variations of the *HIS1* gene in hybrid rice parents provide a non-transgenic approach that can be widely applied to address the seed purity issue of 2-line hybrid rice.

## Methods

### Plant Materials and Genotyping

A total of 631 *indica* rice accessions commonly used in Chinese rice breeding were used in this study, including 221 conventional lines, 213 hybrid rice 3-line restorers, 59 hybrid rice 2-line restorers, 87 CMS, and 51 P/TGMS (supplementary Table 1). Details of these rice accessions are listed in the “China Rice Data Center” (http://www.ricedata.cn/variety/index.htm). *Indica* IR64 (BBC-sensitive) and *japonica* Nipponbare (BBC-resistant) were used as controls. An F2 population derived from a cross between Nongxiang42 (BBC resistant with *HIS1* H2) and Xianhui207 (BBC sensitive with *HIS1* H3) was used to test the relationship of the T1510G mutation and the susceptibility to BBC. Genomic DNA fragments from the start codon to the stop codon of the *HIS1* gene were obtained by high-fidelity PCR. The PCR products were directly sequenced by TsingKe Biotech (Changsha, China). Genotyping with an InDel marker specific to the 28-bp deletion was performed as previously described (Maeda et al. 2019). A dCAPS marker specific to the T1510G mutation in the *HIS1* gene was designed with the online software dCAPS Finder 2.0 (http://helix.wustl.edu/dcaps/dcaps.html), and the restriction enzyme *Dde1* was obtained from New England Biolabs (PCR primers are listed in Table S2). Genotypes of the *HIS1* gene in 3000 rice accessions and the classification information of these rice accessions were obtained from the Rice SNP-Seek Database (Alexandrov et al. 2015) (https://snp-seek.irri.org).

### Chemicals

The herbicide benzobicyclon (BBC) (trade name “Cong Feng”, 25% active ingredients) was obtained from SDS Biotech (Tokyo, Japan). The herbicide mesotrione (trade name “Qian Xiao”, 10% active ingredients) was obtained from FengShan Corp. (Jiangsu, China).

### Detection of the Susceptibility of Rice Accessions to Herbicide

All procedures were carried out as described in a previous study (Maeda et al. 2019), with some modifications: 493 rice seeds were soaked for 2 days and germinated for 1 day at 37 °C. About 50 mL of vermiculite was mixed with half-strength MS culture medium (no sugar and agar) (obtained from BaiSi Biotech, Hangzhou, China) and placed in a plastic cup, and the germinated rice seeds were placed on the surface of the vermiculite. Seedlings were grown at 28 °C for 8 d with 16 h of light and 8 h of dark per day. After the first leaves had expanded, water or water containing herbicide was sprayed on the leaves of the rice seedlings. Seedling growth was assessed after 10 or 15 d. Each rice accession was tested three times for sensitivity to BBC or mesotrione. Compared with the control, unaffected seedling growth was designated as grade 0; seedling growth that was slightly weakened was designated as grade 1; seedlings with an obvious dwarfing phenomenon were designated as grade 2; seedlings without obvious bleaching but that had started to die were designated as grade 3; seedlings that had bleached completely were designated as grade 4; and seedlings that died were designated as grade 5. Grades 0 and 1 were designated as completely resistant; grades 2 and 3 were designated as moderately resistant and moderately susceptible, respectively; and grades 4 and 5 were designated as completely susceptible. The 2-line hybrid rice variety Mingliangyou143 and its female parent MingS and male parent P143 were planted by direct seeding in a paddy field. After the second leaves had expanded (10 d after seeding), BBC (300 g of active ingredient/ha) was sprayed on the leaves of the rice seedlings. Seedling growth was assessed after 15 d.

### Expression Profile of *HIS1* Genes

Total RNA was extracted from rice seedlings using an RNeasy Plant Mini Kit (Qiagen). Semi-quantitative PCR was conducted as previously described (Maeda et al. 2019) with the rice *actin* gene as an internal control. PCR amplification of the *HIS1* gene was 35 cycles, and that of the *actin* gene was 28 cycles. Quantitative PCR analysis was performed with the TransScript Green One-Step qRT-PCR System (TransGen Biotech). The specific primers CCJQ-F and CCJQ-R were used for detection of the *HIS1* and ActinQ-F and ActinQ-R for the reference gene *actin*.

### Alignment of Protein Sequences

Amino acid sequence alignment of 91 experimentally characterized 2OGDs in plants and 114 putative 2OGDs in rice (Kawai et al. 2014) was performed using the CLUSTALX2 algorithm. Motif discovery on protein was performed using the software MEME Suite 5.2.0 (Bailey and Elkan 1994) (http://meme-suite.org/tools/meme).

### Protein Homology Model Building

A homology model for HIS1 was generated using Swiss-model (https://swissmodel.expasy.org), and images were generated using PyMOL (Yuan et al. 2016).

## Supplementary Information

**Additional file 1: Table S1**. The genetic variations of *HIS1* in *indica* rice accessions.

**Additional file 2: Table S2**. PCR primers used in this study.

**Additional file 3: Fig. S1**. PCR-based genotyping for the 28-bp deletion in *indica* accessions (partially). M: DL2000 DNA marker; lane 1–24: IR64, Nipponbare, Bing 1A, Digu, Yuetai A, 9311, Zhenxian 97A, Fengyuan A, Mianhui 725, II-32A, Tianfeng A, Fenghuazan, Yuanhui 2 hao, Yuetai B, IR36, Gui 99, Fengyuan A, Shuhui 498, Miyang 46, IR64, D62A, G46A, CDR22, Wufeng A. **Fig. S2**. Pfam motifs of *HIS1* and amino acid variation of haplotypes. The shaded parts are predicted amino acids due to the 28-bp deletion in H5. **Fig. S3**. Protein sequence alignment of 2OGDs. (A) Protein sequence alignment and conserved site analysis of 91 experimentally characterized 2OGDs in plants. (B) Protein sequence alignment and conserved site analysis of 114 putative 2OGDs in rice. **Fig. S4**. RNA expression of *HIS1* in some accessions harboring H1, H2, H3, and H5. The qRT-PCR results were normalized with the *actin* reference gene. Error bars represent the standard error of the mean (SEM) of three replicates. **Fig. S5**. The T1510G mutation is completely co-segregated with the susceptibility to BBC (partially). Rice accessions Nongxiang42 and Xianhui207 carry *HIS1* haplotypes H2 and H3, respectively. The upper electrophoretic band represents the PCR products of *HIS1,* and the lower band shows the PCR products treated with the restriction enzyme *Dde1*. **Fig. S6**. BBC is effective when the seeds are mixed. (A). BBC can effectively kill hybrid sterile lines (MingS) without harming hybrids (Mingliangyou143). The red arrow indicates the sterile line that was killed. (B). Random selection of 10 normal and severely affected seedlings. Through the detection of 28-bp deletion primers, it was found that the normal materials were all in a heterozygous state, and the sterile line materials lacking 28-bp were killed.

## Data Availability

All data supporting the conclusions of this article are provided within the article (and its additional files).

## Abbreviations

bTH: β-triketone herbicide
BBC: Benzobicyclon
TPR: Puddled-transplanted rice
DSR: Direct seeding of rice
HPPD: 4-hydroxyphenylpyruvate dioxygenase
*HIS1*: *HPPD INHIBITOR SENSITIVE 1*
ALS: Acetolactate synthetase
ACCase: Acetyl-CoA carboxylase
P/TGMS: Photoperiod- and thermo-sensitive genic male sterile
CMS: Cytoplasm male sterile
