# Supplementary material for: Exploring Natural Allelic Variations of the β-Triketone Herbicide Resistance Gene HIS1 for Application in indica Rice and Particularly in Two-Line Hybrid Rice
Source: Rice (N Y). 2021 Jan 7;14:7. doi: 10.1186/s12284-020-00448-7 (PMC7790941; doi:10.1186/s12284-020-00448-7)
Supplement: Supplementary file 3 — Additional file 3: Fig. S1. PCR-based genotyping for the 28-bp deletion in indica accessions (partially). M: DL2000 DNA marker; lane 1–24: IR64, Nipponbare, Bing 1A, Digu, Yuetai A, 9311, Zhenxian 97A, Fengyuan A, Mianhui 725, II-32A, Tianfeng A, Fenghuazan, Yuanhui 2 hao, Yuetai B, IR36, Gui 99, Fengyuan A, Shuhui 498, Miyang 46, IR64, D62A, G46A, CDR22, Wufeng A. Fig. S2. Pfam motifs of HIS1 and amino acid variation of haplotypes. The shaded parts are predicted amino acids due to the 28-bp deletion in H5. Fig. S3. Protein sequence alignment of 2OGDs. (A) Protein sequence alignment and conserved site analysis of 91 experimentally characterized 2OGDs in plants. (B) Protein sequence alignment and conserved site analysis of 114 putative 2OGDs in rice. Fig. S4. RNA expression of HIS1 in some accessions harboring H1, H2, H3, and H5. The qRT-PCR results were normalized with the actin reference gene. Error bars represent the standard error of the mean (SEM) of three replicates. Fig. S5. The T1510G mutation is completely co-segregated with the susceptibility to BBC (partially). Rice accessions Nongxiang42 and Xianhui207 carry HIS1 haplotypes H2 and H3, respectively. The upper electrophoretic band represents the PCR products of HIS1, and the lower band shows the PCR products treated with the restriction enzyme Dde1. Fig. S6. BBC is effective when the seeds are mixed. (A). BBC can effectively kill hybrid sterile lines (MingS) without harming hybrids (Mingliangyou143). The red arrow indicates the sterile line that was killed. (B). Random selection of 10 normal and severely affected seedlings. Through the detection of 28-bp deletion primers, it was found that the normal materials were all in a heterozygous state, and the sterile line materials lacking 28-bp were killed. [file 12284_2020_448_MOESM3_ESM.docx]

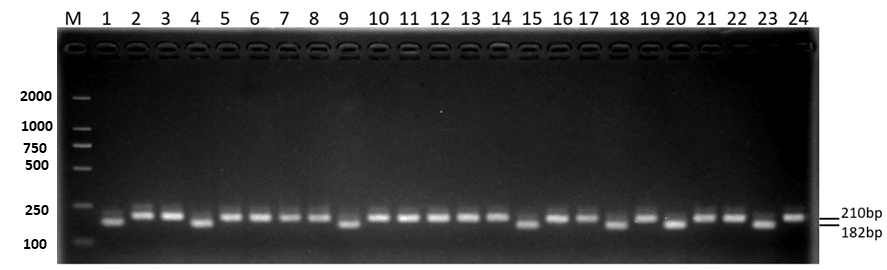


Fig. S1. PCR-based genotyping for the 28-bp deletion in *indica* accessions (partially)

M: DL2000 DNA marker; lane 1-24: IR64, Nipponbare, Bing 1A, Digu, Yuetai A, 9311, Zhenxian 97A, Fengyuan A, Mianhui 725, II-32A, Tianfeng A, Fenghuazan, Yuanhui 2 hao, Yuetai B, IR36, Gui 99, Fengyuan A, Shuhui 498, Miyang 46, IR64, D62A, G46A, CDR22, Wufeng A


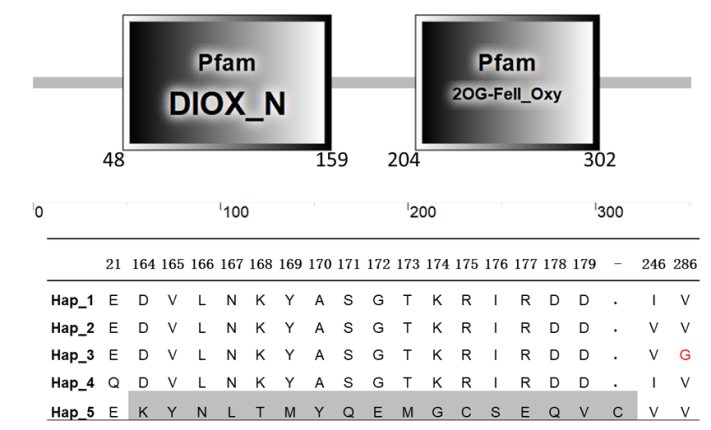


Fig. S2. Pfam motifs of *HIS1* and amino acid variation of haplotypes

The shaded parts are predicted amino acids due to the 28-bp deletion in H5.


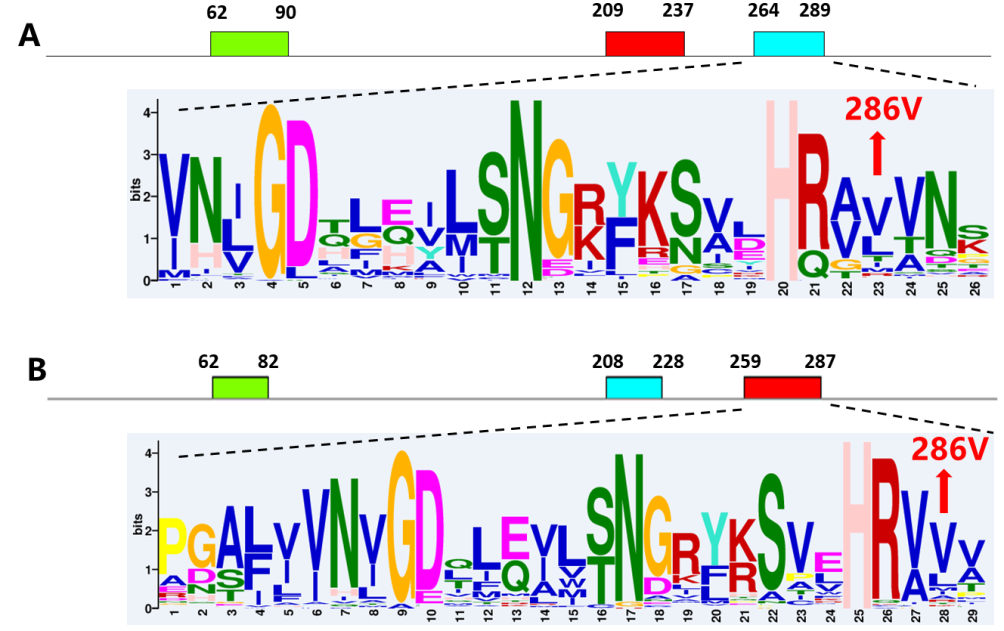


Fig. S3. Protein sequence alignment of 2OGDs

(A) Protein sequence alignment and conserved site analysis of 91 experimentally characterized 2OGDs in plants. (B) Protein sequence alignment and conserved site analysis of 114 putative 2OGDs in rice.


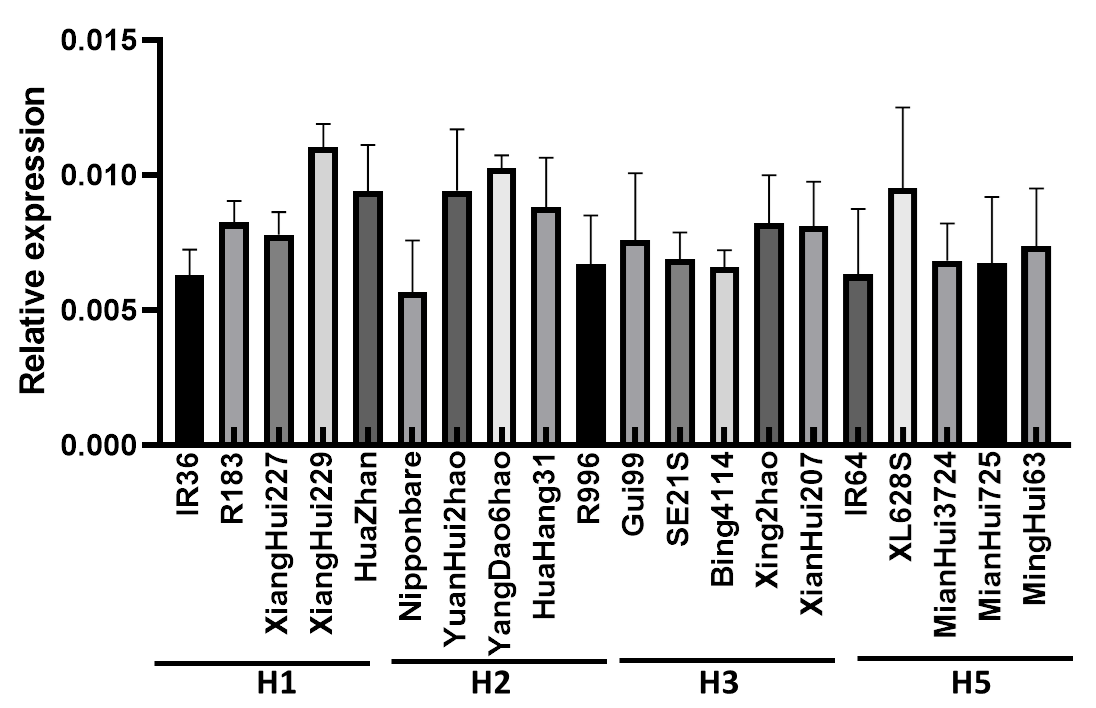


Fig. S4. RNA expression of *HIS1* in some accessions harboring H1, H2, H3, and H5.

The qRT-PCR results were normalized with the *actin* reference gene. Error bars represent the standard error of the mean (SEM) of three replicates.


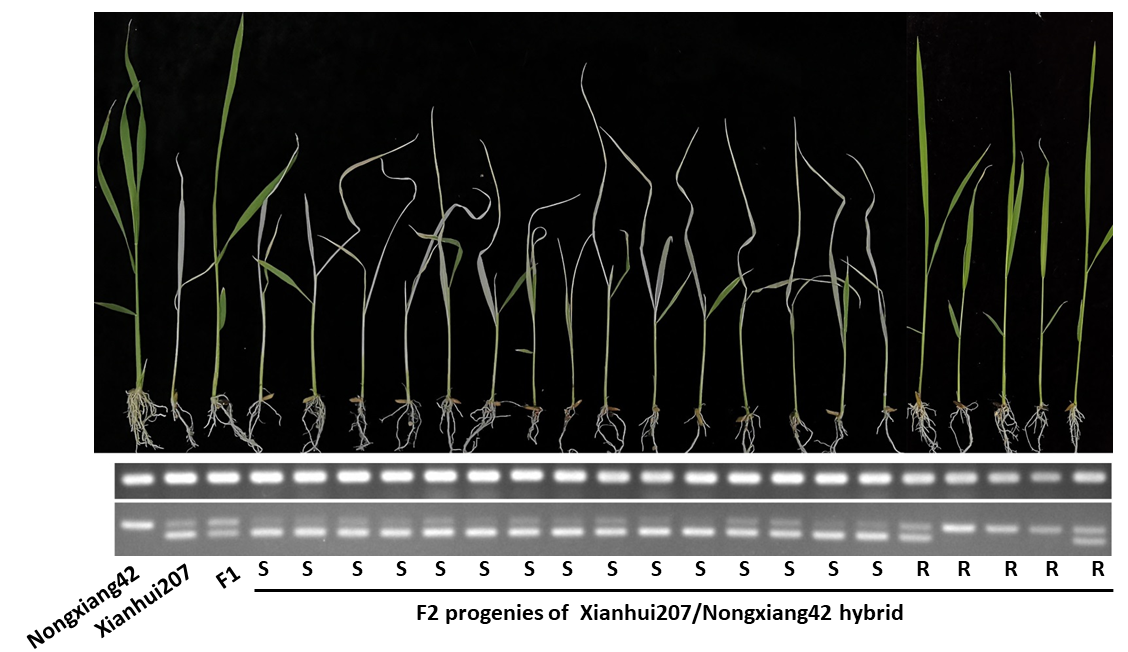


Fig. S5. The T1510G mutation is completely co-segregated with the susceptibility to BBC (partially)

Rice accessions Nongxiang42 and Xianhui207 carry *HIS1* haplotypes H2 and H3, respectively. The upper electrophoretic band represents the PCR products of *HIS1,* and the lower band shows the PCR products treated with the restriction enzyme *Dde1*.


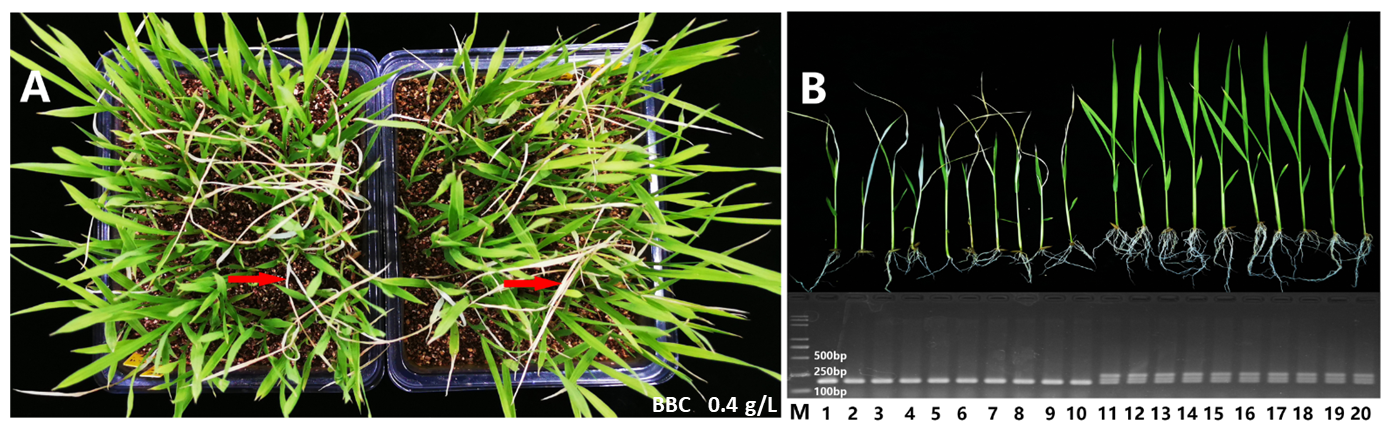


Fig. S6. BBC is effective when the seeds are mixed

(A). BBC can effectively kill hybrid sterile lines (MingS) without harming hybrids (Mingliangyou143). The red arrow indicates the sterile line that was killed. (B). Random selection of 10 normal and severely affected seedlings. Through the detection of 28-bp deletion primers, it was found that the normal materials were all in a heterozygous state, and the sterile line materials lacking 28-bp were killed.
